# Colchicine Attenuates Microvascular Obstruction after Myocardial Ischemia-Reperfusion Injury by Inhibiting the Proliferation of Neutrophil in Bone Marrow

**DOI:** 10.1007/s10557-023-07528-y

**Published:** 2023-12-08

**Authors:** Ying Tan, Xue Bao, Yuyu Li, Guo Song, He Lu, Xuan Sun, Rong Gu, Lina Kang, Biao Xu

**Affiliations:** 1 Department of Cardiology, Nanjing Drum Tower Hospital, Affiliated Hospital of Medical School, Nanjing University, Nanjing, 210008 Jiangsu China; 2 Key Laboratory of Remodeling-Related Cardiovascular Diseases (Ministry of Education) and Beijing Institute of Heart, Lung, and Blood Vessel Diseases, Beijing Anzhen, Hospital Affiliated to Capital Medical University, Beijing, 100029 China; 3 Department of Cardiology, Nanjing Drum Tower Hospital Clinical College of Nanjing Medical University, Nanjing, 210008 Jiangsu China; 4 Department of Cardiology, Nanjing Drum Tower Hospital Clinical College of Nanjing University of Chinese Medicine, Nanjing, 210008 Jiangsu China

**Keywords:** Myocardial ischemia/reperfusion injury, Microvascular obstruction, Colchicine, NETs, S100A8/A9

## Abstract

**Purpose:**

Complete and rapid recanalization of blood flow by percutaneous coronary intervention (PCI) is the most effective intervention for patients with ST-segment elevation myocardial infarction (STEMI). However, myocardial ischemia/reperfusion (I/R) injury leads to microvascular obstruction (MVO), limiting its efficacy. Colchicine can reduce myocardial I/R injury, but its effect on MVO is unclear. Hence, this study aimed to assess the role and mechanism of colchicine on MVO.

**Methods:**

Clinical data on STEMI patients with PCI were collected and risk factors related to MVO were analyzed. The rat myocardial I/R model was established to evaluate the MVO by thioflavin S staining. The myocardial I/R model of mice was treated with PBS or colchicine at the reperfusion. The effect of colchicine on cardiomyocyte apoptosis after I/R was evaluated by TUNEL and expression of cleaved caspase-3. ROS levels were detected in H9c2 cells to evaluate the colchicine effect on myocardial oxidative stress. Moreover, the mechanism through which colchicine attenuated MVO was examined using flow cytometry, WB, ELISA, immunohistochemistry, bioinformatics analysis, and immunofluorescence.

**Results:**

Multivariate analysis showed that elevated neutrophils were associated with extensive MVO. Colchicine could attenuate MVO and reduce neutrophil recruitment and NETs formation after myocardial I/R. In addition, colchicine inhibited cardiomyocyte apoptosis in vivo and ROS levels in vitro. Furthermore, colchicine inhibited neutrophil proliferation in the bone marrow (BM) by inhibiting the S100A8/A9 inflammatory signaling pathway.

**Conclusions:**

Colchicine attenuated MVO after myocardial I/R injury by inhibiting the proliferation of neutrophils in BM through the neutrophil-derived S100A8/A9 inflammatory signaling pathway.

**Supplementary Information:**

The online version contains supplementary material available at 10.1007/s10557-023-07528-y.

## Introduction

Acute ST-segment elevation myocardial infarction (STEMI) remains a leading cause of death and disability worldwide [1]. Since the beginning of the twenty-first century, with the development and popularization of primary percutaneous coronary intervention (PCI), an increasing number of patients have received interventional treatment in the acute phase of myocardial infarction. While the mortality rate in the acute phase has decreased significantly, the long-term prognosis remains worrying related to myocardial ischemia/reperfusion (I/R) injury. Restoration of myocardial perfusion and the subsequent rapid activation of myocardial inflammation and necrosis cause the myocardial I/R injury [2]. After undergoing PCI, a subset of patients continue to experience inadequate blood supply to the myocardial tissue, a condition known as “no-reflow” after I/R injury. This contributes to the development of microvascular obstruction (MVO) [3, 4]. In recent years, MVO has been recognized as an independent risk factor for the prognosis of STEMI patients. Several clinical trials have shown that patients with extensive MVO after PCI have worse long-term outcomes and higher mortality [5, 6]. Therefore, the development of effective therapies to attenuate MVO is an urgent clinical need.

Colchicine is an anti-inflammatory drug that has recently shown potential in the treatment of coronary heart disease (CHD). Results from the low-dose colchicine (LoDoCo) [7] study and the colchicine cardiovascular outcome trial (COLCOT) [8] have shown that long-term colchicine treatment significantly reduces the risk of secondary ischemic cardiovascular events. Previous clinical data have shown that colchicine, when administered 6 to 24 h before PCI, could reduce periprocedural myocardial injury [9]. Colchicine was found to improve pre-PCI coronary flow reserve (CFR) and resistive reserve ratio with lower levels of troponin and inflammatory mediators, but there was no difference in “no-reflow” [9]. Whether colchicine can attenuate MVO in STEMI patients after myocardial I/R injury is unclear and its mechanism still needs to be further elucidated.

An elevated neutrophil count has been confirmed to be associated with increased infarct size (IS) and decreased left ventricular ejection fraction (LVEF) [10]. Our clinical study showed a significant correlation between neutrophil count and the severity of MVO. Neutrophils can exacerbate the formation of MVO through the formation of neutrophil extracellular traps (NETs) [11]. NETs are a network of DNA histones and proteins released by activated neutrophils [12–14]. Therefore, we hypothesized that inhibiting neutrophils and NETs can attenuate MVO after myocardial I/R injury.

In this study, we found that the colchicine could inhibit neutrophil recruitment and NETs after myocardial I/R. In addition, we observed that colchicine could inhibit cardiomyocyte apoptosis after myocardial I/R and reduce ROS levels after hypoxia/reoxygenation (H/R). Notably, colchicine demonstrated the ability to attenuate MVO. Based on these findings, we proposed the hypothesis that colchicine can attenuate MVO after myocardial I/R injury by inhibiting neutrophil proliferation in the BM through the S100A8/A9 inflammatory signaling pathway. This study will provide a potential therapeutic strategy for attenuating myocardial I/R injury, particularly in the context of MVO.

## Materials and Methods

### Participants and Data Collection

#### Patients

A total of 115 patients with STEMI who underwent emergency PCI at Nanjing Drum Tower Hospital from October 2021 to December 2022 were selected for the study. Blood samples were collected for routine blood tests before PCI, and cardiac magnetic resonance (CMR) and echocardiography were performed within 1 week after PCI. Inclusion criteria of patients were: (1) consistent with the diagnostic criteria for acute STEMI [2], (2) primary PCI within 12 h after onset, (3) age ≤ 85 years old, and (4) informed consent was signed. This study was approved by the Ethics Committee of Nanjing Drum Tower Hospital. The approval number was 2021-531-02.

#### CMR Assessment

All patients underwent CMR examination within 1 week after PCI using the Philips 3.0T Ingenia magnetic resonance imaging system. The patients were positioned in a supine position, and the breath-holding sequence was scanned over 12 cardiac cycles with a breath-holding time of 12–17 s. Gadolinium was then administered via a peripheral vein for first-pass perfusion scans, and myocardial delayed gradient echo inversion recovery sequence enhancement scans were performed 10 min after injection (TR: 6 ms, TE: 3 ms, FA: 25°, TI: 260–350 ms, shot duration: 100–125 ms, voxel: 1.6 mm × 1.9 mm × 8 mm). CMR images were analyzed by experienced radiologists. The area of IS was shown as hyperintensity in images, and MVO was described as hypointense areas in the area of IS in late-gadolinium enhancement (LGE) CMR images. The representative CMR-LGE images are shown in Supplementary Fig. 1. Both IS and MVO were expressed as their percentage of left ventricle (LV).

### Animal Experiments and Ethics Statement

All animal experiments were approved by the Institutional Ethics Committee of Nanjing Drum Hospital (2021AE02005) and followed the guidelines outlined in the Guidelines for the Care and Use of Laboratory Animals published by the National Institutes of Health. Male Sprague−Dawley (SD) rats (n = 12, weighing 220−250 g, 7−8 weeks) and C57BL/6 mice (n = 50, weighing 20−25 g, 7−8 weeks) were purchased from the Center for Model Animals, Nanjing University. Animals were placed in 22 °C and 65–70% humidity chambers for 12 h light–dark cycles and fed a standard laboratory diet with free access to food and water. In our study, the rat experiment was only used to observe the MVO area since MVO, which stands for microvascular obstruction, is defined as a very small area that is not easily observable. The rat heart volume is larger than that of mice, making it more convenient to observe the MVO size in hearts. The other animal experiments in this study were all conducted on mice. Rats or mice were divided into three groups—sham group, I/R+ PBS group, and I/R + colchicine group. At the end of the study, all animals were given 1.5% isoflurane inhalation anesthesia followed by euthanasia by cervical dislocation.

Rats or mice were used to establish the myocardial I/R model. They were anesthetized by inhalation of isoflurane (1.5–2%) and ventilated with room air using a ventilator. A thoracotomy was performed at the fourth intercostal space to expose the heart and left anterior descending coronary artery (LAD). The rats’ LAD was ligated with a 4–0 silk suture and the mice’s LAD was ligated with a 7–0 silk thread and released after 45 min to restore blood perfusion [15]. For colchicine treatment, rats or mice were administered colchicine once (intraperitoneal injections, 0.1 mg/kg) (C3915, Sigma-Aldrich, USA) immediately after reperfusion. The dose of colchicine was determined according to a previously published study [16]. The artery was separated without ligation in the sham operation group.

### Assessment of Microvascular Obstruction (MVO) Size

After 24 h of reperfusion, the rats (n = 4 in sham, I/R+PBS, I/R+ colchicine group separately) were placed under general anesthesia with isoflurane, and the limbs were immobilized in a supine position on an animal surgical plate. The thoracic cavity was reopened along the original incision to expose the heart, and the rat’s aorta was carefully separated to expose it; 1 mL thioflavin S (4% solution, Sigma Aldrich) was injected via the aorta to delineate the no-reflow zone. The heart was quickly excised and cut crosswise into 1 mm-thick slices by heart slice mold (1 mm spacing). Slices were exposed to UV light (302 nm) using a UV transilluminator for digital imaging. Light blue indicates myocardial uptake after infusion of thioflavin-S into the area at risk, whereas dark blue indicates lack of perfusion which was delineated as areas of MVO by Image J software [17]. Percentage MVO was expressed as a percentage of left ventricular (LV) [18].

### Flow Cytometry Analysis

#### Blood and Bone Marrow Neutrophils

Mice (n = 4 each group) were euthanized at 12 h after reperfusion and their ocular venous blood was collected in EDTA tubes, and erythrocytes were lysed using red blood cell (RBC) lysis buffer (Invitrogen, USA). They were washed, rinsed, and resuspended in phosphate-buffered saline (PBS) or FACS buffer for fluorescent antibodies (anti-mouse PE-labeled antibody against Ly6G (1:50, 12-9668-80, Invitrogen, USA) and FITC-labeled antibody against CD45 (1:50, 11-0451-81, Invitrogen, USA)) staining. The femur and tibia of each mouse were isolated and washed, and their ends were cut to expose the marrow cavity. The BM was washed directly with cold PBS using a 40 μm nylon mesh strainer. After the supernatant was aspirated, the RBCs were lysed in the RBC lysis buffer. Cells were then resuspended in FACS buffer and subjected to fluorescent antibody (anti-mouse PE-labeled antibody against Ly6G (1:50, 12-9668-80, Invitrogen, USA) and a FITC-labeled antibody against CD45 (1:50, 11-0451-81, Invitrogen, USA)) staining, washed, and resuspended in FACS buffer for flow cytometry analysis.

#### Heart and Spleen Neutrophils

The hearts (n = 4 each group) were collected at 24 h after reperfusion and spleen tissues (n = 4 each group) were collected at 12 h after reperfusion after the euthanization of the mice. A single-cell suspension of the tissues was obtained by using a gentle MACS^TM^ Dissociator (Miltenyi Biotec). Then, the samples were stained with the antibodies (anti-mouse PE-labeled antibody against Ly6G (1:50, 12-9668-80, Invitrogen, USA) and FITC-labeled antibody against CD45 (1:50, 11-0451-81, Invitrogen, USA)), washed, and resuspended in FACS buffer for flow cytometry analysis.

Flow cytometry was carried out on an FACS Aria flow cytometer (BD Bioscience, USA), and data were analyzed with FlowJo software (TreeStar, Ashland, OR, USA).

### In Vivo Proliferation Assay (EdU)

For proliferation studies, the I/R+ ABR-215757 treated mice group was built. The S100A8/A9 inhibitor (ABR-215757) was administered orally once (10 mg/kg) 4 h before LAD ligation, and the treatment was continued (10 mg·kg^−1^·d^−1^) in drinking water until termination mice [19]. Mice (n = 4 in sham, I/R+PBS, I/R+ colchicine, I/R+ S100A8/A9 inhibitor group separately) were injected with 0.2 mg of 5-ethyny l-2’-deoxyuridine (EdU) for 12–14 h via intraperitoneal injection before euthanization. In preparation for flow cytometry, after 12 h of reperfusion, cell populations were immunostained CD45^+^Ly6G^+^ and the incorporation of EdU was quantified using Click-iT^TM^ Plus EdU flow cytometry assay kit (Molecular Probes, Eugene OR) according to the manufacturer’s instructions. Proliferation was quantified and expressed as a percentage of EdU^+^ cells [19].

### Cell Culture

Primary neutrophils were collected from bone marrow as previously described [20]. Briefly, bone marrow cells were collected from the femurs and tibias of 4-week-old mice by flushing with RPMI 1640 medium containing 10 U/mL heparin, 50 U/mL penicillin, and 50 g/mL streptomycin. Then, cells were treated with RBC lysis buffer and filtered through a 70 μm mesh. The cell pellet was resuspended in 200 μL buffer per 5×10^7^ cells. Primary neutrophils were sorted from the cells by magnetic beads according to the manufacturer’s instructions (130-097-658, Miltenyi Biotec, Germany). After washing with PBS, primary neutrophils were resuspended in RPMI 1640 supplemented with 10% fetal bovine serum (FBS), 50 U/mL penicillin, and 50 g/mL streptomycin, followed by inoculation in a humidified atmosphere of 5% CO_2_ and 95% O_2_ at 37 °C [21]. To verify whether colchicine inhibits the S100A8/S100A9 signaling pathway in neutrophils, recombinant mouse S100A8/S100A9 (2 µg/ml, R&D Systems, MN, USA) was added to the culture medium in primary neutrophils for 8–12 h with or without colchicine (10 μmol/L, C3915, Sigma-Aldrich, USA) pretreatment [19, 21]. NLRP3 expression was measured using western blotting.

### Preparation of Hypoxia/Reoxygenation (H/R) Cell Model and Measurement of Intracellular ROS

The H9c2 cardiomyocytes (Rat cardiomyocytes; American Typer Culture Collection, Manassas, USA) were maintained in DMEM supplemented with 10% FBS and 50 U/mL penicillin, and 50 g/mL streptomycin. The culture conditions contained 95% O_2_ and 5% CO_2_ at 37 °C. Cells in the control group were cultured under normal conditions. The H/R group cells were placed in a three-gas incubator (1% O_2_, 94% N_2_, and 5% CO_2_) under hypoxia for 12 h. Then, the cells were cultured with normal DMEM in a carbon dioxide incubator (5% CO_2_ and 95% O_2_) for 6 h for reoxygenation. The H/R group was treated with colchicine (5 nmol/L) or PBS and exposed to H/R, simultaneously [22].

Myocardial oxidative stress was measured using a reactive oxygen species assay kit (ROS Assay Kit) (S0033S, Beyotime, China) [16]. After 6 h of reoxygenation, the medium of H9c2 cells was changed to a serum-free medium containing DCFH-DA (10 μM). After incubation at 37 °C for 30 min, images were taken by fluorescence microscope (Leica Thunder, Germany) [16]. ROS were green (excitation wavelength 488 nm, emission wavelength 535 nm) in cells [22].

### Enzyme-linked immunosorbent Assays (ELISA)

Peripheral blood samples were collected at 6, 12, 24, and 48 h of reperfusion after myocardial I/R for ELISA detection. EDTA anticoagulant blood samples (n = 4 each group) were collected and incubated at room temperature on a shaker for 30 min. Platelet-depleted plasma was collected after centrifugation at 2400 g for 20 min at 4 °C, and interleukin-1β (IL-1β), interleukin-6 (IL-6), tumor necrosis factor-alpha (TNF-α), and S100A8/A9 protein levels were measured using commercial ELISA kits according to the manufacturer’s instructions (Sbjbio-Z/E, Nanjing, China).

### Immunostaining Fluorescence

After 24 h reperfusion of the myocardial I/R model, fresh heart tissue (n = 4 each group) was embedded in an optimal cutting temperature compound (OCT) at –80 °C for 2h, cut into 6mm sections, and stored at –80 °C. Sections were washed with PBS, blocked with goat serum for 1h, and incubated overnight at 4 °C with primary antibodies, marker of Nets: Ly6G^+^(1:200, ab25377, Abcam, UK) MPO^+^(1:100, 14569, CST, USA) CitH4^+^(1:100, 07–596, Sigma-Aldrich, USA), three co-localized, markers of cardiomyocyte: α-Actinin (Sarcomeric) (α-Actinin) (1:200, A7811, Sigma-Aldrich, USA). After washing three times with PBS, the cells were incubated with secondary antibodies (Alexa Flour 488, 1:500, 4412, CST, Alexa Fluor 555,1:500, 4413, CST, and Alexa Flour 647 1:500, 4414, CST, USA) or (Cy™3 AffiniPure Donkey Anti-Rabbit IgG (H+L), 1:400, 711-165-150, Jackson ImmunoResearch, USA) for 2 h at room temperature in the dark. Nuclei were counterstained with DAPI. Samples were sealed with 50% glycerol. Images were captured using a Leica imaging system.

### TUNEL Assay

TUNEL assay was used to detect cardiomyocyte apoptosis in myocardial tissue. The sections were examined with a TUNEL assay kit (Beyotime Biotechnology, Shanghai, China). Images of the infarct area were acquired in each section using an OLYMPUS microscope (Japan). Image J software was used to count the number of TUNEL-positive cells in each of the total number of myocardial nuclei, and to calculate the percentage of apoptotic cardiomyocytes [23].

### Immunohistochemistry

After the mice (n = 3 each group) were euthanized after 24 h reperfusion, the femur, tibia, and fibula were harvested for histological studies. Immunohistochemical staining was used to detect the expression of IL-1RI, IL-1RII, and IL-1Ra in the BM. Paraffin sections were deparaffinized and rehydrated, then deposited and stored at 4 °C overnight before being incubated with primary antibodies IL-1RI (1:50, sc-393998, Santa Cruz, USA), IL-1RII (1:50, sc-376247, Inc), IL-1RII (1:50, sc-376247, Santa Cruz, USA), and IL-1Ra (1:50, sc-374084, Santa Cruz, USA). The next day, the corresponding secondary antibody was applied at 20 to 25 °C for about 50 min, followed by the prepared 3,3’-diaminobenzidine (DAB) substrate and hematoxylin. The results were evaluated by two qualified pathologists. Motic Images Plus version 2.0 system (Motic China Group Co, Xiamen, China) was used for image acquisition and the ImageJ analysis system was quantified.

### Western Blotting

Cultured cells or heart tissues in mice euthanized after 24 h reperfusion were homogenized in a lysis buffer containing a mixture of protease and phosphatidase inhibitors. The homogenate was denatured in a loading buffer at 95 °C for 10 min; 30 ug of protein was loaded onto SDS-PAGE gels and transferred to PVDF membranes. Subsequently, cell membranes were blocked with TBST containing BSA (5%), followed by incubation with specific antibodies (caspase-3(1:1000, 14220, CST, USA), cleaved caspase-3 (1:1000, 9664, CST, USA), Tubulin (1:1000, 11224-1-AP, Proteintech, USA), NLRP3(1:1000, 15101, CST, USA), and GAPDH (1:1000, 5174, CST, USA)) overnight at 4 °C. Finally, cells were incubated with horseradish peroxidase-labeled anti-rabbit immunoglobulin G. The signals were detected with an electrochemiluminescence (ECL) system and quantified by scanning densitometry with an ImageJ analysis system.

### Bioinformatics Analysis

The datasets were collected from the Gene Expression Omnibus (GEO) database. The initial work was to identify differentially expressed genes (DEGs) and then find common DEGs for Colchicine, NETs, MVO, and I/R. Gene Ontology (GO) analysis of selected differential genes was performed using the online tool DAVID (https://david.ncifcrf.gov/), and the Kyoto Encyclopedia of Genomes (KEGG) pathway enrichment analysis of differential genes was performed using the KOBAS online analysis database (http://kobas.cbi.pku.edu.cn/). The results were presented as bar graphs and bubble plots, respectively. Then the STRING online database protein–protein interaction (PPI) network was used to analyze the association of DEGs and to identify the interacting molecules of colchicine in myocardial I/R injury.

### Statistical Analysis

The Clinical Research Section used the Kolmogorov–Smirnov test to test for normality. Continuous normally distributed data are represented as mean ± standard deviation (SD) and are compared using the unpaired Student *t*-test. Nonnormal distribution variables are reported as median (interquartile range) and compared using the Mann–Whitney *U* test. Categorical variables are expressed as frequency and percentage and are appropriately studied by either chi-square or Fisher’s exact test. The univariate analysis was divided into two groups: extensive and no or mild MVO groups; we selected 2.6% as the segmentation value, divided patients into two groups, included all baseline variables of *P* <0.05 in univariate analysis into the logistic regression model, calculated their odds ratio and 95% confidence interval, and *P*-values <0.05 were considered statistically significant. All statistical analyses were performed using the IBM SPSS software package (IBM SPSS Statistics 25).

#### Basic Experimental Section

All values are expressed as mean ±SD or median, with interquartile ranges as appropriate. For data with normal distribution and homogeneity of variance, the unpaired t-test was used to compare the two groups. For the comparison of multiple samples at the same time point, the one-way ANOVA was used to confirm the existence of differences between groups, and then Tukey’s test was used to compare the means of each group. For the comparison of multiple samples at multiple time points, two-way ANOVA was used to confirm the existence of differences between groups, and Bonferroni’s test was used to compare the means of each group. The difference in *P*-value < 0.05 was considered statistically significant. Statistical analysis was carried out using GraphPad Prism 9.0.

## Results

### Neutrophils Correlated with the Severity of MVO after Myocardial I/R

The data of 115 patients with STEMI who underwent emergency PCI for the first time in Nanjing Drum Tower Hospital from October 2021 to December 2022 were collected. The CMR examination was performed 1 week within the emergency PCI to analyze the area of MVO and the ratio of MVO to LV. They were classified as extensive MVO group (MVO ≥ 2.6%LV) and no or mild MVO group (MVO < 2.6%LV) according to a previous study [5] (Fig. S1). The percentage of neutrophils (%) in blood in the extensive MVO group was significantly higher than that in the no or mild MVO group (Table 1). The extensive MVO group has a larger area of IS and lower LVEF than the no or mild MVO group. There was no statistical difference between the two groups in terms of other baseline characteristics, such as risk factors of CHD, previous cerebral infarction, previous angina, heart rate, blood pressure, body mass index (BMI), Killip class, first medical contact to guidewire passage time (FMC-wire time) and medications before or after infarct in Table 1. Fourteen factors in univariate analysis were chosen for multivariate logistic regression analysis according to previous studies [10, 24, 25]. The result showed that the elevated neutrophils was independently associated with extensive MVO (*P* = 0.028) (Table 2). Our sensitivity analysis showed that *P*-value of neutrophils (%), per 1SD change after additional adjustment for intravenous diuretic was 0.0285, and the *p* value was 0.0374 after additional adjustment for LVEF (%) in CMR, indicating that the number of neutrophils was independently associated with extensive MVO after controlling for IS and LVEF.

**Table 1:** Baseline demographic and clinical characteristics

	No or mild MVO (n = 79)	Extensive MVO (n = 36)	*p* value
MVO (%/LV)	0.7±0.7	4.73±2.53	–
Neutrophil (%)	73 ± 12.9	78.1 ± 9.9	**0.0399**
Age, y	60.9 ± 12.9	62.5 ± 12.7	0.5045
Male, No. (%)	64 (81)	33 (89.2)	0.1561
BMI	25.1 ± 2.9	24.4 ± 3.1	0.2363
Risk factors [No. (%)]			
Hypertension	40 (50.6)	18 (48.7)	0.9498
Diabetes	13 (16.5)	5 (13.5)	0.7257
Smoking	40 (50.6)	24 (64.9)	0.1111
Family history of CHD	2 (2.53)	2 (5.41)	0.4233
Previous cerebral infarction, No. (%)	4 (5.06)	1 (2.7)	0.5829
Previous angina, No. (%)	20 (25.3)	9 (24.3)	0.9711
Heart rate, bpm	81.2 ± 15.9	82.1 ± 14.4	0.7765
Systolic blood pressure, mmHg	126.6 ± 20.3	125.4 ± 21	0.9093
Diastolic arterial pressure, mmHg	82.9 ± 12.5	79.5 ± 12.3	0.172
Mean arterial pressure, mmHg	97.46 ± 15.1	94.8 ± 15.2	0.418
Killip class	1.11 ± 0.45	1.3 ± 0.66	0.1237
Anterior MI, No. (%)	35 (44.3)	21 (56.8)	0.1648
IS (%/LV)	19.5 ± 10.8	27.5 ± 11.1	0.0004
LVEF (%)	47.3 ± 4.86	43.4 ± 4.94	0.0003
FMC-wire time (h)	1.49 ± 0.39	2.07 ± 0.93	0.081
Medications before PCI			
Beta-blockers	5 (6.33)	2 (5.56)	0.8723
Ace-inhibitors	4 (5.06)	1 (2.78)	0.5829
Statins	1 (1.27)	0 (0)	0.9536
Medications post PCI			
Clopidogrel	18 (22.8)	6 (16.7)	0.4559
Ticagrelor	63 (79.8)	30 (83.3)	0.6507
Intravenous diuretic	4 (5.06)	6 (16.7)	0.0521
Nitrates	6 (7.59)	4 (11.1)	0.5372

*p* = 0.039 (*p*< 0.05) Neutrophils (%) in blood in the extensive MVO group was significantly higher than that in the no or mild MVO group

**Table 2:** Multivariable logistic regression analysis

	OR (95% CI)	*p* value
Neutrophils (%), per 1SD change	2.26 (1.18, 5.12)	**0.028**
Age, y	1.02 (0.98, 1.07)	0.334
Male, No. (%)	7.89 (1.43, 60.7)	0.028
BMI	0.82 (0.66, 0.99)	0.056
Hypertension	1.41 (0.50, 4.11)	0.522
Diabetes	1.31 (0.31, 5.12)	0.701
Previous cerebral infarction, No. (%)	0.18 (0.01, 1.84)	0.195
Previous angina, No. (%)	1.43 (0.45, 4.55)	0.5441
Smoking	2.41 (0.81, 7.84)	0.125
Family history of CHD	1.9 (0.11, 35.75)	0.657
Systolic blood pressure, mmHg	1.01 (0.98, 1.04)	0.500
Killip class on admission	2.35 (0.84, 7.48)	0.109
Anterior MI, No. (%)	1.42 (0.54, 3.84)	0.478
IS (%/LV)	1.08 (1.03, 1.14)	0.002

*p* = 0.028 (*p*< 0.05) Elevated neutrophils was independently associated with extensive MVO

### Colchicine Attenuated MVO in Rats and Inhibited NETs in Mice after Myocardial I/R Injury

**Fig. 1:**
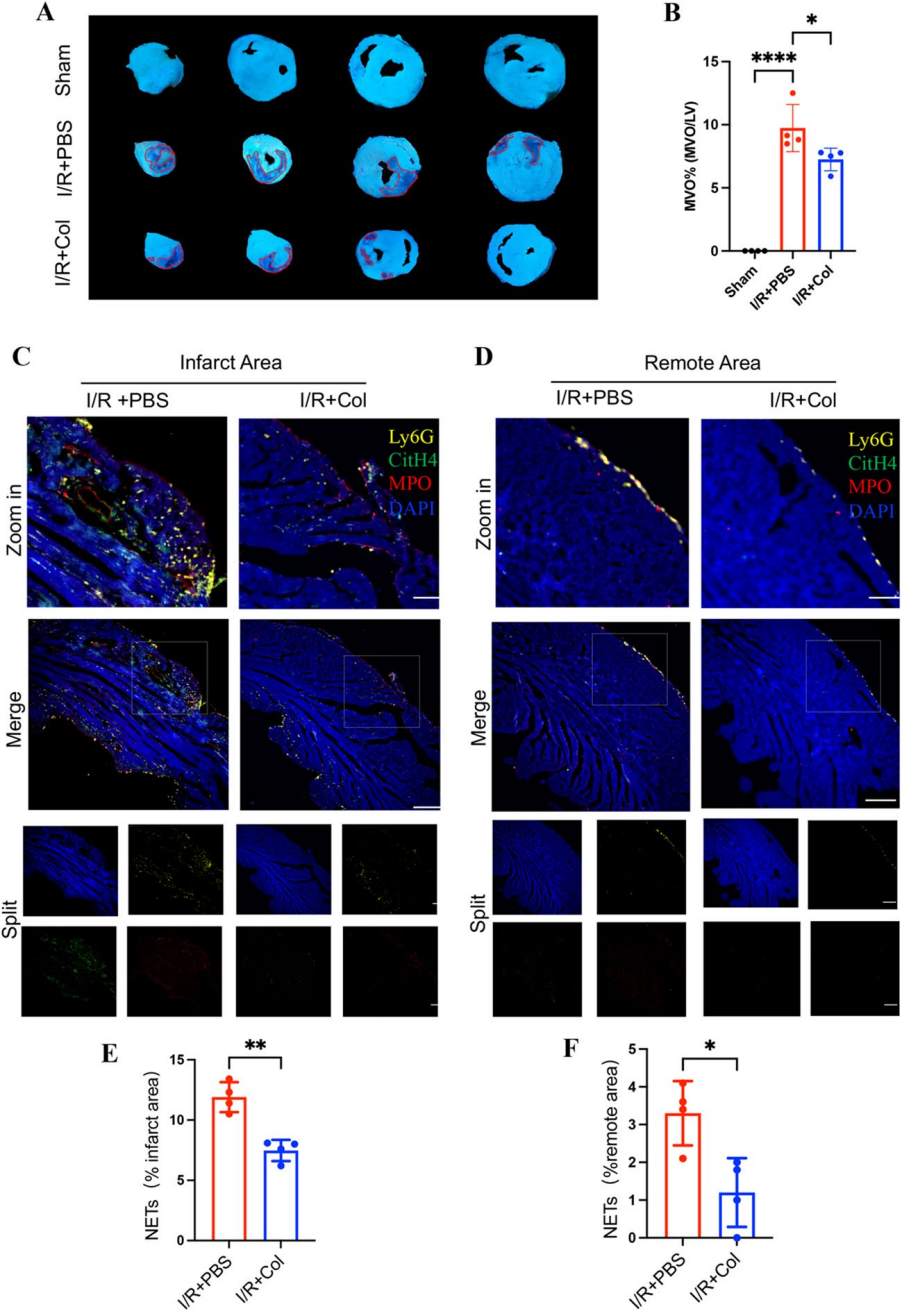
Colchicine alleviated MVO in rats and inhibits NETs in mice after myocardial I/R injury. (**A**) Representative image of thioflavin-S-stained hearts of myocardial I/R-injured rats treated with PBS or colchicine for 24 h. The area of the red trace is the MVO (scar bar = 5mm). (**B**) MVO% (MVO/LV) in the ischemic heart 24 h after myocardial I/R (n = 4 each group) (not normalized to infarct size). (**C**) Representative immunofluorescence images of NETs in the myocardial infarct area and (**D**) remote area staining with Ly6G (yellow), CitH4 (citrullinated histone 4, green), MPO (myeloperoxidase, red), and DAPI (blue) (scale bar = 200 μm). (**E**) Semi-quantification analysis of NETs of infarct and (**F**) remote areas (n = 4 each group). Data are shown as mean ± SD. *P < 0.05, **P < 0.01, ****P <0.0001. Col, colchicine

To determine whether colchicine could attenuate MVO after I/R injury, we used the ligation model of the left anterior descending artery in SD rats for 45 min of ischemia and 24 h of reperfusion. PBS or colchicine was given immediately on reperfusion. After 24 h, myocardial tissue was taken for thioflavin S staining. It was found that the MVO area of the colchicine treatment group was lower than that of the I/R+PBS group (Fig. 1A, B), suggesting that colchicine could attenuate MVO after myocardial I/R injury, where MVO % was not normalized to infarct size here. Following myocardial infarction, neutrophils are the first inflammatory cells to arrive at the injured myocardium and can activate inflammation by releasing NETs [26]. NETs enhance inflammation and participate in myocardial I/R injury [27]. A previous study reported that colchicine suppressed NET formation in patients with acute coronary syndrome post-PCI by restoring cytoskeletal dynamics [28]. Therefore, we aimed to clarify whether colchicine attenuated MVO through the inhibition of NETs. The results of mice experiments showed that colchicine was able to attenuate NETs in the ischemic and peri-ischemic regions. This was confirmed by immunofluorescence localization of NETs (Ly6G^+^CitH4^+^MPO^+^) [29, 30] in myocardial tissue 24 h after myocardial I/R (Fig. 1C–F).

### Colchicine Inhibited Cardiomyocyte Apoptosis in Mice after Myocardial I/R and Reduced ROS Levels In Vitro

**Fig. 2:**
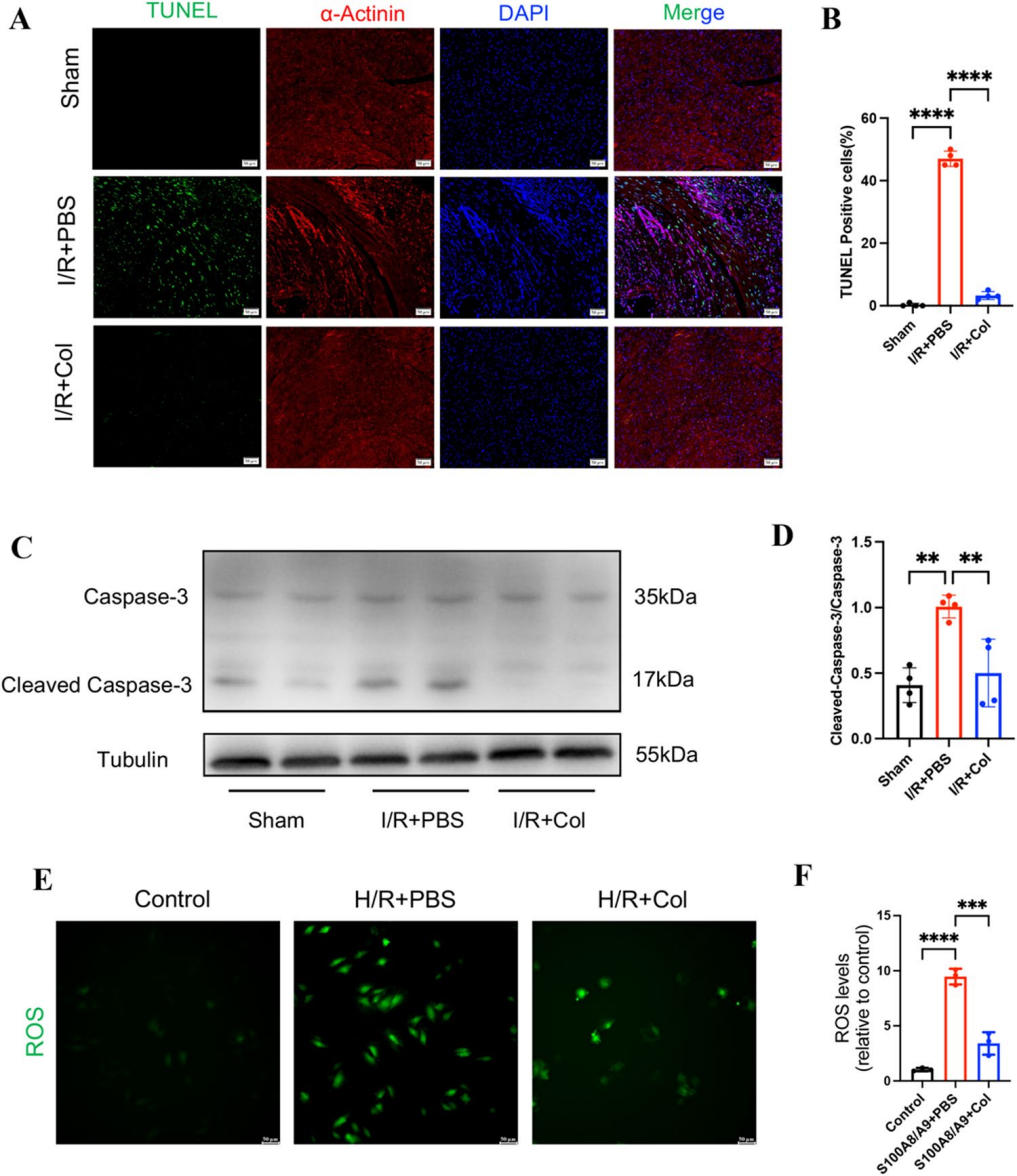
Colchicine inhibited cardiomyocyte apoptosis in mice after myocardial I/R and reduced ROS levels in vitro. (**A**) Representative immunofluorescence images of cardiac tissues staining with TUNEL (green), α-Actinin (red), and DAPI (blue) in the infarct hearts of the mice at 1 day after myocardial I/R (scar bar = 50 μm). (**B**) Quantitative analysis of TUNEL (green) positive cells in cardiomyocyte (n = 4 each group). (**C**) Representative western blot images of total caspase-3 and cleaved caspase-3 in the myocardial infarct area. (**D**) Statistical analysis of cleaved caspase-3 protein expression in the myocardial infarct area (n = 4 each group). (**E**) Representative immunofluorescence images of ROS in H9c2 cells treated by H/R with PBS or colchicine pretreatment (scale bars = 50 μm). (**F**) Statistical analysis of ROS levels in H9c2 cells (n = 3 each group). Data are shown as mean ± SD. ns, not significant. **P < 0.01, ***P < 0.001, ****P < 0.0001. Col, colchicine

Next, we found that colchicine treatment could inhibit cardiomyocyte apoptosis as indicated by decreased TUNEL staining in the infarct hearts of the mice 1 day after myocardial I/R (Fig. 2A, B). Meanwhile, apoptosis was assessed by the expression of cleaved caspase-3 and caspase-3 in cardiomyocytes and the result showed that colchicine inhibited the protein levels of cleaved caspase-3 after myocardial I/R (Fig. 2C–D). Additionally, in order to determine the effect of colchicine on myocardial oxidative stress, a hypoxia/reoxygenation (H/R) model was established in H9c2 cells to mimic myocardial I/R, and ROS level was measured by ROS Assay Kit. The result showed that colchicine could inhibit the ROS level increased by H/R (Fig. 2E–F). Taken together, these data suggested that colchicine inhibited cardiomyocyte apoptosis in vivo and reduced ROS levels in vitro.

### Colchicine Reduced Neutrophils after Myocardial I/R Injury and Inhibited Inflammatory Factors in Mice after Myocardial I/R

**Fig. 3:**
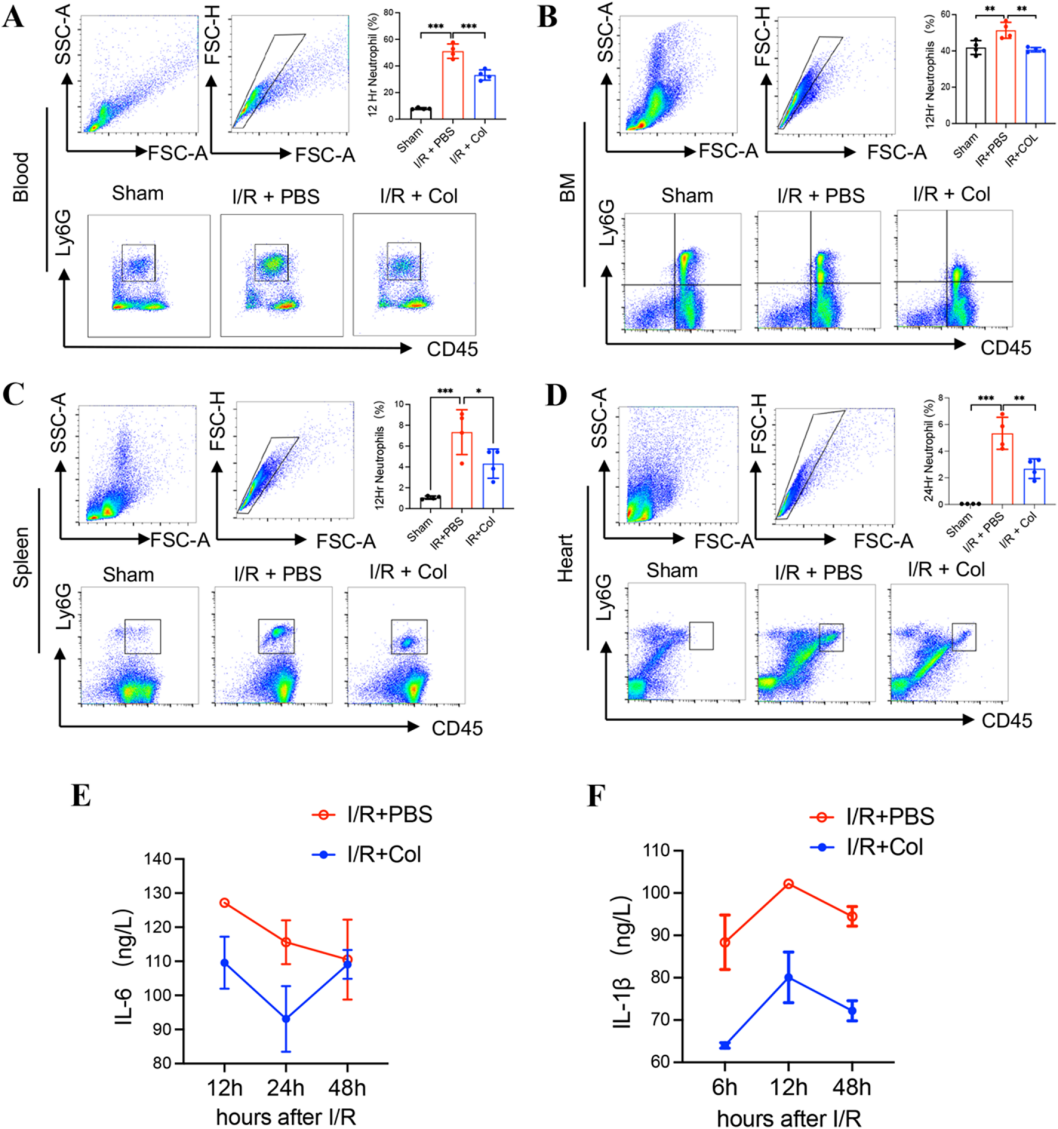
Colchicine reduced the recruitment of neutrophils and inhibited inflammatory factors in mice after myocardial I/R. (**A–D**) Representative flow cytometry plots and statistical analysis of CD45^+^Ly6G^+^ neutrophils in the blood, BM, spleen, and heart at 12, 12, 12, and 24 h after myocardial I/R separately (n = 4 each group). (**E**) Quantification of IL-6 protein levels (by ELISA) in the plasma of mice at 12, 24, and 48 h after myocardial I/R (n = 4 each group). (**F**) Quantification of IL-1β protein levels (by ELISA) in the plasma at 6, 12, and 48 h after myocardial I/R (n = 3 each group). Data are shown as mean ± SD. *P < 0.05, **P < 0.01, ***P < 0.001. Col, colchicine

In our study, we found that neutrophil counts were correlated with the severity of MVO, and colchicine could inhibit NETs in hearts after myocardial I/R. Therefore, we focused on the changes in neutrophils, which were identified as the target cells of colchicine. Flow cytometry analysis revealed that colchicine significantly inhibited myocardial I/R-induced neutrophil (CD45^+^Ly6G^+^) accumulation in the blood, BM, spleen, and heart (Fig. 3A–D). These data indicated that colchicine could reduce neutrophils in important organs, particularly inhibiting the recruitment of neutrophils in hearts after myocardial I/R injury. As colchicine is an anti-inflammatory drug, we then investigated the levels of inflammatory cytokines in circulation after treatment with colchicine in the myocardial I/R model. The expression of inflammatory factors in serum was measured at 6, 12, 24, and 48 h after myocardial I/R in mice. Compared with the I/R+PBS group, the circulating levels of IL-1β, and IL-6 were significantly downregulated in the colchicine treatment (Fig. 3E, F).

### Colchicine Inhibited Neutrophil Proliferation in the BM after Myocardial I/R

**Fig. 4:**
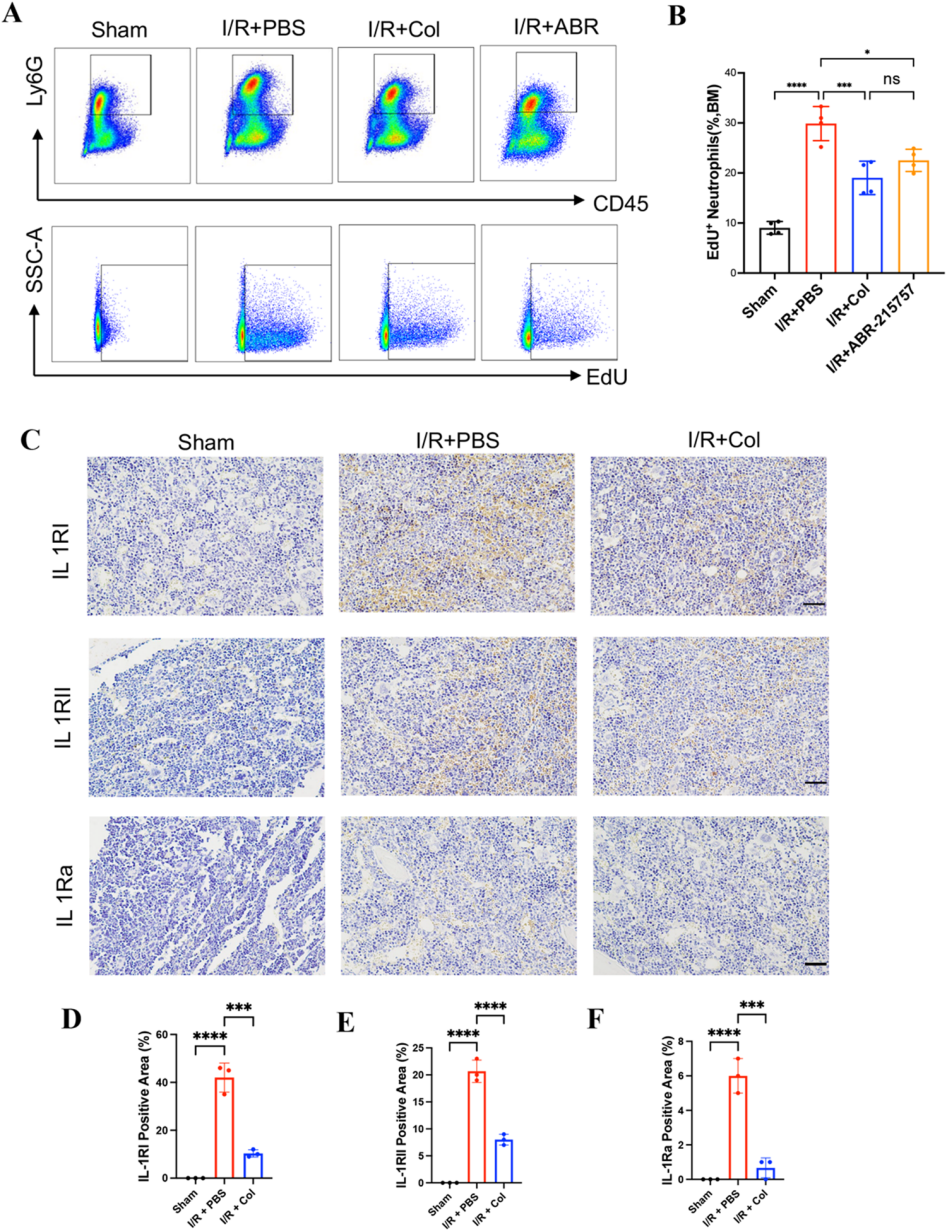
Colchicine inhibited neutrophil proliferation and Il-1β receptor expression in the BM of mice after myocardial I/R. (**A**) Representative flow cytometry plots and (**B**) statistical analysis of CD45^+^Ly6G^+^ neutrophils (as assessed by EdU incorporation) in the BM of mice at 12 h after myocardial I/R (n = 4 each group). (**C**) Representative immunohistochemistry images of IL-1RI, IL-1RII, and IL-1Ra in BM of mice at 12 h after myocardial I/R (scale bar = 20 μm). (**E–F**) Quantification of IL-1RI, IL-1RII, and IL-1Ra positive area in BM of mice at 12 h after I/R (n = 3 each group). Data are shown as mean ± SD. ns, not significant, *P < 0.05, ***P < 0.005, ****P < 0.001. BM, bone marrow. Col, colchicine. ABR-215757, S100A8/A9 inhibitor

It has been reported that myocardial infarction triggers myelopoiesis, resulting in heightened production of neutrophils and an increase in blood neutrophils levels due to the mass exodus of neutrophils from their reservoirs in the BM [19]. To investigate whether colchicine has the ability to inhibit neutrophil proliferation in the bone marrow, we assessed EdU incorporation. The result showed that colchicine could inhibit the proliferation of neutrophils in BM at 12 h after myocardial I/R (Fig. 4A, B). Similarly, immunohistochemistry of the BM showed that colchicine could inhibit the Il-1β receptor (IL-1R) in the BM, including both IL-1RI and IL-1RII receptors (Fig. 4C–E). IL-1 receptor antagonist (IL-1Ra) is a cytokine that inhibits the binding of Il-1β to the interleukin receptor by neutralizing the activity of IL-1. IL-1Ra helps to suppress immune and inflammatory responses [31]. IL-1Ra was also inhibited by colchicine in BM after myocardial I/R (Fig. 4C,F). Therefore, it is speculated that colchicine reduced the number of circulating neutrophils and their recruitment to the injured myocardium by inhibiting neutrophil proliferation in the BM after myocardial I/R.

### Colchicine Inhibited Proliferation of Neutrophil by Inhibiting S100A8/A9-NLRP3/IL-1β/IL-1R Inflammatory Pathway

**Fig. 5:**
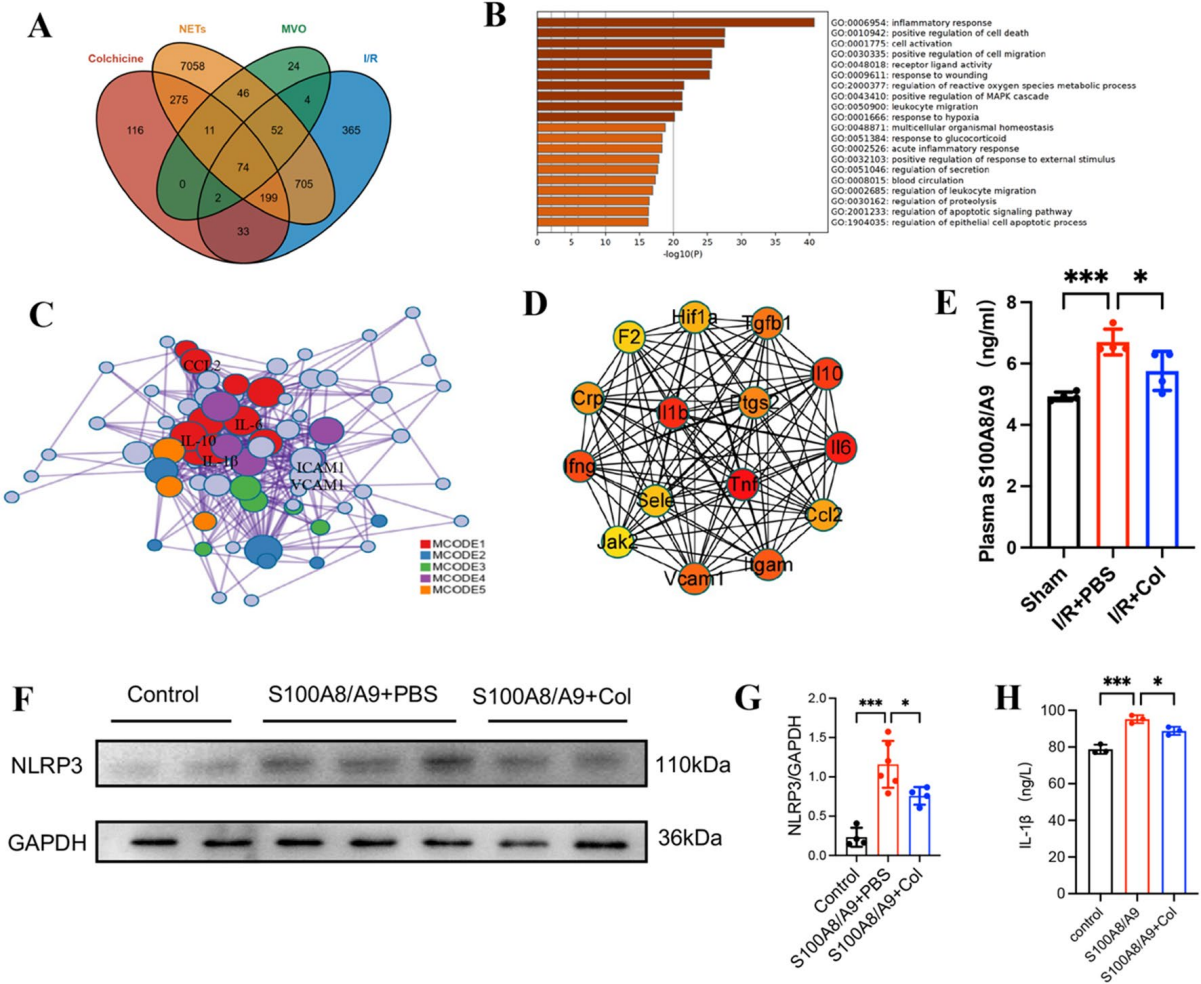
Colchicine inhibited neutrophil proliferation in the BM through the S100A8/A9-NLRP3/IL-1β/IL-1R pathway. (**A**) The Venn diagram shows the overlap of the four datasets, and colchicine, NETs, MVO, and I/R were selected. (**B**) Bar chart of enriched items from 74 genes, colored with *P*-values. (**C**) Network of enriched items. (**D**) Interaction network of protein targets. (**E**) Quantification of S100A8/A9 protein levels (by ELISA) in the plasma of mice at 12 h after myocardial I/R injury (n = 4 each group). (**F**) Representative western blot images and (**G**) statistical analysis of NLRP3 protein expression in primary neutrophils (n = 4–6 each group), GAPDH shown as the loading control. (**H**) Quantification of IL-1β protein levels (by ELISA) in primary neutrophils (n = 3 each group). Data are shown as mean ± SD. *P < 0.05, ***P < 0.005. Col, colchicine

To investigate the possible mechanism that colchicine attenuated MVO and inhibited NETs after myocardial I/R injury, we used the keywords “Colchicine, NETs, MVO, and I/R” to find differentially expressed genes (DEGs) in the National Center for Biotechnology Information (NCBI)-Gene Expression Omnibus (GEO) database, and set the *P*-value and fold change (FC) to screen for differential genes. The differences were considered statistically significant when *P* < 0. 05, ∣log2FC∣ > 2. The results showed that (Fig. 5A) 74 genes were found common in all DEGs. The enrichment analysis ranked the inflammatory response-related gene pathways first (Fig. 5B–C, S2B). The protein target interaction network expressed in genes was obtained using the STRING database, and the results showed that IL-1β may be a key candidate gene for colchicine in the MVO action pathway (Fig. 5D), which needs further experimental validation.

**Fig. 6:**
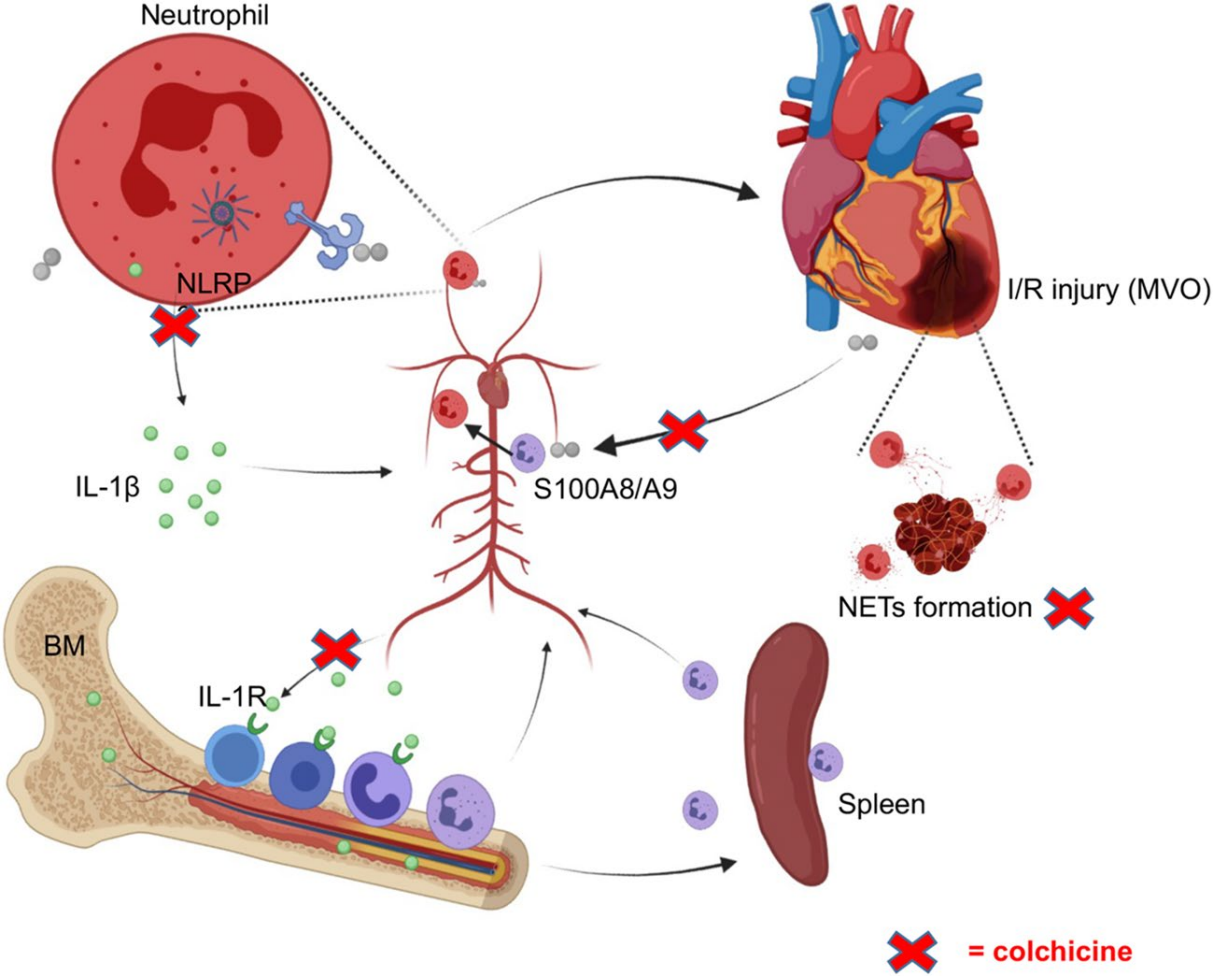
Schematic representation of the signaling pathway that colchicine inhibited neutrophil proliferation in the BM. In response to myocardial I/R injury, circulating neutrophils and NETs formation are attracted to the areas of MVO, where they are rapidly primed and release S100A8/A9, stimulating the expression of the NLRP3 inflammasome in circulating neutrophils and secretes IL-1β. IL-1β interacts with its receptor (IL-1R) in the bone marrow. The BM is stimulated to produce more neutrophils to the circulation and recruited to the MVO area, thus aggravating myocardial injury. Colchicine can inhibit the recruitment of neutrophils in circulation and inhibit the neutrophils proliferation in BM by inhibiting the S100A8/A9 signaling pathway, thereby attenuating MVO after myocardial I/R injury

To further examine the mechanism through which colchicine inhibits the proliferation of neutrophils in BM, we explored the role of the S100A8/A9-NLRP3-IL-1β signaling axis, which has been reported to regulate granulopoiesis and improve cardiac function in mouse myocardial infarction models [19]. We measured the concentration of S100A8/A9 in serum by ELISA in the mice myocardial I/R model at different time points. It was found that colchicine inhibited the S100A8/A9 protein level in blood at 12 h after myocardial I/R (Fig. 5E). The S100A8/A9 protein level reached the peak 12 h after reperfusion (Fig. S2A), which was consistent with a previous study [19]. It is widely believed that the S100A8/A9-NLRP3-IL-1β signaling axis is housed mainly in cardiac neutrophils [19, 32–34]. Thus, we hypothesized that colchicine might inhibit the proliferation of neutrophils in BM through the S100A8/A9 signaling pathway in neutrophils. ABR-215757, an S100A8/A inhibitor, was also found to inhibit the proliferation of neutrophils in BM after myocardial I/R (Fig. 4A, B). To further investigate the effect of colchicine on the S100A8/A9 signaling pathway, we conducted in vitro experiments. Primary neutrophils were isolated from BM by magnetic beads, and recombinant mouse S100A8/S100A9 was administrated to mimic the release of S100A8/A9 after myocardial I/R. The primary neutrophils were then cultured in the presence or absence of colchicine for 8–12 h with or without colchicine. Western blot showed that colchicine inhibited S100A8/A9-induced NLRP3 expression in neutrophils (Fig. 5F–G). The results of ELISA showed that colchicine treatment could reduce the protein levels of IL-1β (Fig. 5H) and TNF-α (Fig. S2C), which was consistent with the results of the myocardial I/R model in mice. Collectively, these data indicated that colchicine could attenuate MVO after myocardial I/R injury by inhibiting the proliferation of neutrophils in BM through the S100A8/A9-NLRP3/IL-1β pathway in neutrophils, as shown in Fig. 6.

## Discussion

In the present study, we found that colchicine attenuated MVO and reduced neutrophil recruitment and NETs formation after myocardial I/R. In addition, colchicine inhibited cardiomyocyte apoptosis in vivo and ROS levels in vitro. The new finding from our study was that colchicine attenuated MVO by inhibiting neutrophil proliferation in BM through the S100A8/A9 inflammatory signaling pathway.

In the early stage of myocardial I/R injury, neutrophils are activated under the action of inflammatory factors and reach the injured heart [35–37]. Neutrophils can be activated through a variety of pathways such as complement reactive oxygen species and cytokines. This promotes neutrophil adhesion, accumulates in damaged tissues, and participates in inflammation and immune response [38–40]. Previous studies have found that S100A8/A9 protein levels were elevated both systemically and at sites of coronary artery occlusion [41, 42]. Plasma S100A8/A9 protein levels have also been associated with left ventricular dysfunction and heart failure and can predict the risk of future cardiovascular disease events [43]. Recent studies have demonstrated that circulating neutrophils were rapidly activated and recruited to the injured myocardium after myocardial infarction, releasing S100A8/S100A9 locally in the ischemic microenvironment. S100A8/S100A9 can bind to TLR-4 on the envelope of resident or recruited neutrophils and activate the downstream NLRP3 inflammasome [44], and then secrete IL-1β [33, 45]. IL-1β can enter the hematopoietic microenvironment of BM with blood circulation, bind to IL-1R on hematopoietic stem cells and hematopoietic progenitor cells in BM and stimulate hematopoietic stem cells and hematopoietic progenitor cells to differentiate into neutrophils and macrophages, which are continuously delivered to the damaged area of the myocardium [19].

MVO is an important manifestation of myocardial I/R injury and indicates poor prognosis in patients with AMI [46]. Elevated and activated neutrophils can not only adhere to the vascular endothelium but also block microcirculation blood vessels [47]. It can also cause damage to the microvascular structure by secreting a large amount of pro-inflammatory substances (such as ROS, proteases, lysosomal enzymes, etc.), causing edema of vascular endothelial cells and forming MVO [48]. In addition, neutrophil-derived NETs can aggravate MVO and produce a no-reflow phenomenon [49].

Colchicine is an effective anti-inflammatory drug. In the early stage of the inflammatory response, it can effectively reduce the chemotactic effect of neutrophils at the site of the inflammatory reaction [8, 50]. In our study, clinical data show that the patients with extensive MVO (≥2.6% LV) had higher baseline neutrophil levels than those without MVO or with mild MVO within 7 days after PCI. Elevated neutrophil levels significantly correlated with increased MVO area in patients with STEMI. The association between the burden of NETs in peripheral blood and MVO in STEMI patients undergoing PCI needs further validation. We first found that colchicine could reduce the MVO area after myocardial I/R injury. Additionally, colchicine could inhibit neutrophil recruitment and NETs after I/R. Furthermore, colchicine inhibited cardiomyocyte apoptosis in vivo and ROS levels in vitro. Importantly, we found that colchicine inhibited neutrophil proliferation in BM. Bioinformatics analysis suggested that IL-1β may be a potential target for colchicine to attenuate MVO after myocardial I/R. Experiments in mice and primary neutrophils further proved that colchicine can attenuate MVO after myocardial I/R injury by inhibiting neutrophil proliferation in BM through the neutrophil-derived alarmins S100A8/A9/NLRP3/IL-1β pathway. Therefore, colchicine can effectively inhibit the inflammatory storm and attenuate MVO in the early stage of myocardial I/R injury.

There are some limitations in this study. Our clinical study is based on the follow-up data of the coronary heart disease intervention database approved by the Ethics Committee of Nanjing Drum Tower Hospital. We have not conducted a prospective pilot study of colchicine. The use of colchicine to improve the prognosis of patients with STEMI remains controversial. Therefore, our aim is to further clarify the role and mechanism of colchicine in MVO, with the results potentially providing a theoretical basis and potential therapies for reducing myocardial I/R injury.

In conclusion, colchicine can effectively inhibit the recruitment of neutrophils and the expression of inflammatory cytokines. Our findings demonstrated that colchicine attenuates MVO after myocardial I/R injury by inhibiting the proliferation of neutrophils in the BM through the S100A8/A9 inflammatory signaling pathway. Several prospective trials on colchicine in patients with AMI are currently underway. Colchicine has the potential to be used in both emergency and routine settings for STEMI patients undergoing PCI. Although further research is still needed, our finding provides evidence for colchicine treatment to improve the prognosis of STEMI patients.

## Supplementary Information

ESM 1 (DOCX 7566 kb)

## Data Availability

The raw data supporting the conclusions of this manuscript will be made available by the authors, without undue reservation, to any qualified researcher.
