# Supplementary material for: Colchicine Attenuates Microvascular Obstruction after Myocardial Ischemia-Reperfusion Injury by Inhibiting the Proliferation of Neutrophil in Bone Marrow
Source: Cardiovasc Drugs Ther. 2023 Dec 8;39(2):259–73. doi: 10.1007/s10557-023-07528-y (PMC11954697; doi:10.1007/s10557-023-07528-y)
Supplement: Supplementary file 1 — (DOCX 7566 kb) [file 10557_2023_7528_MOESM1_ESM.docx]

**
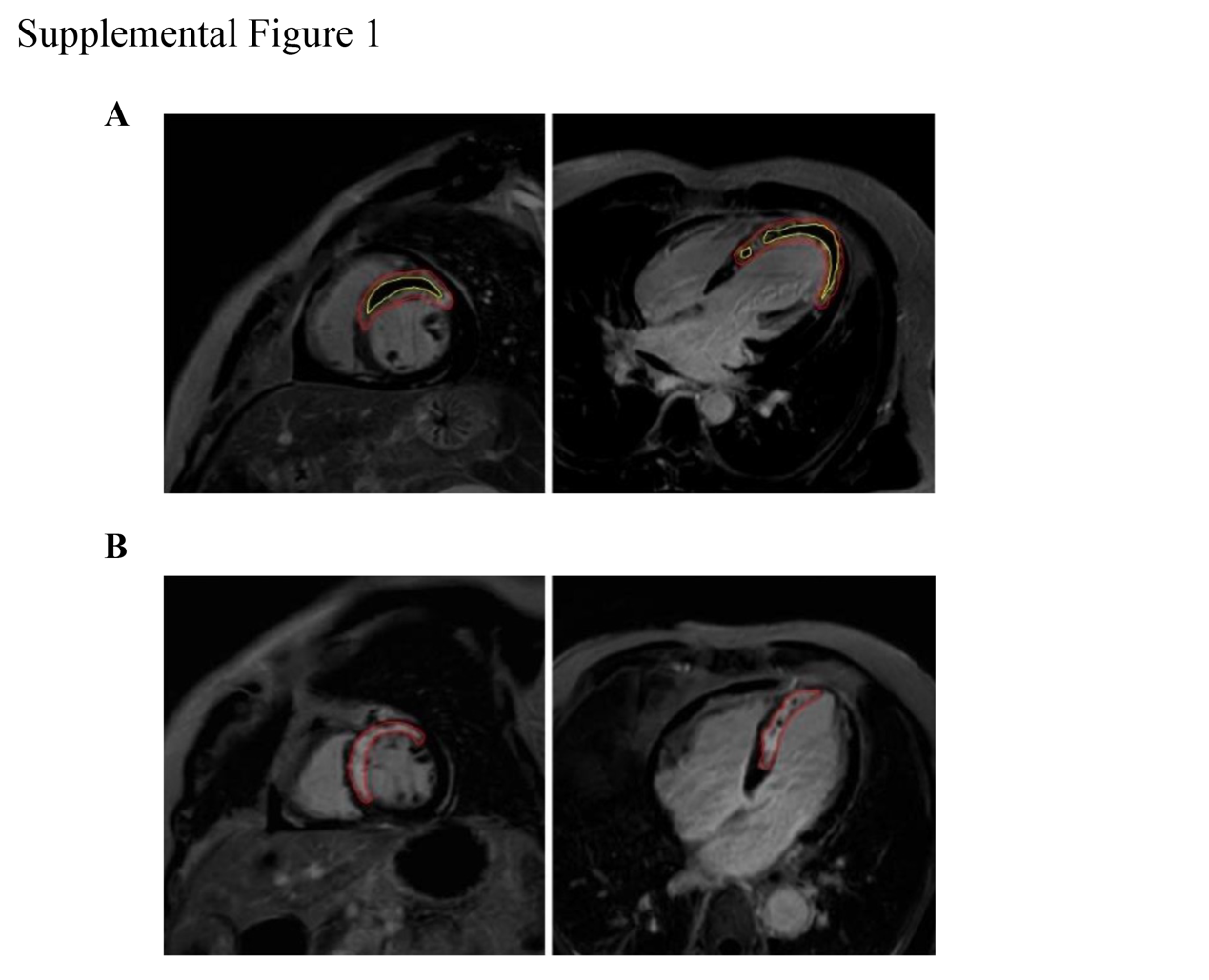
**

**Fig S1** Representative CMR-LGE images of different degrees of MVO in STEMI patients. Red represents the area of infarct size and yellow represents the area of MVO. (**A**) Representative image of extensive MVO. (**B**) Representative image of no or mild MVO.

**
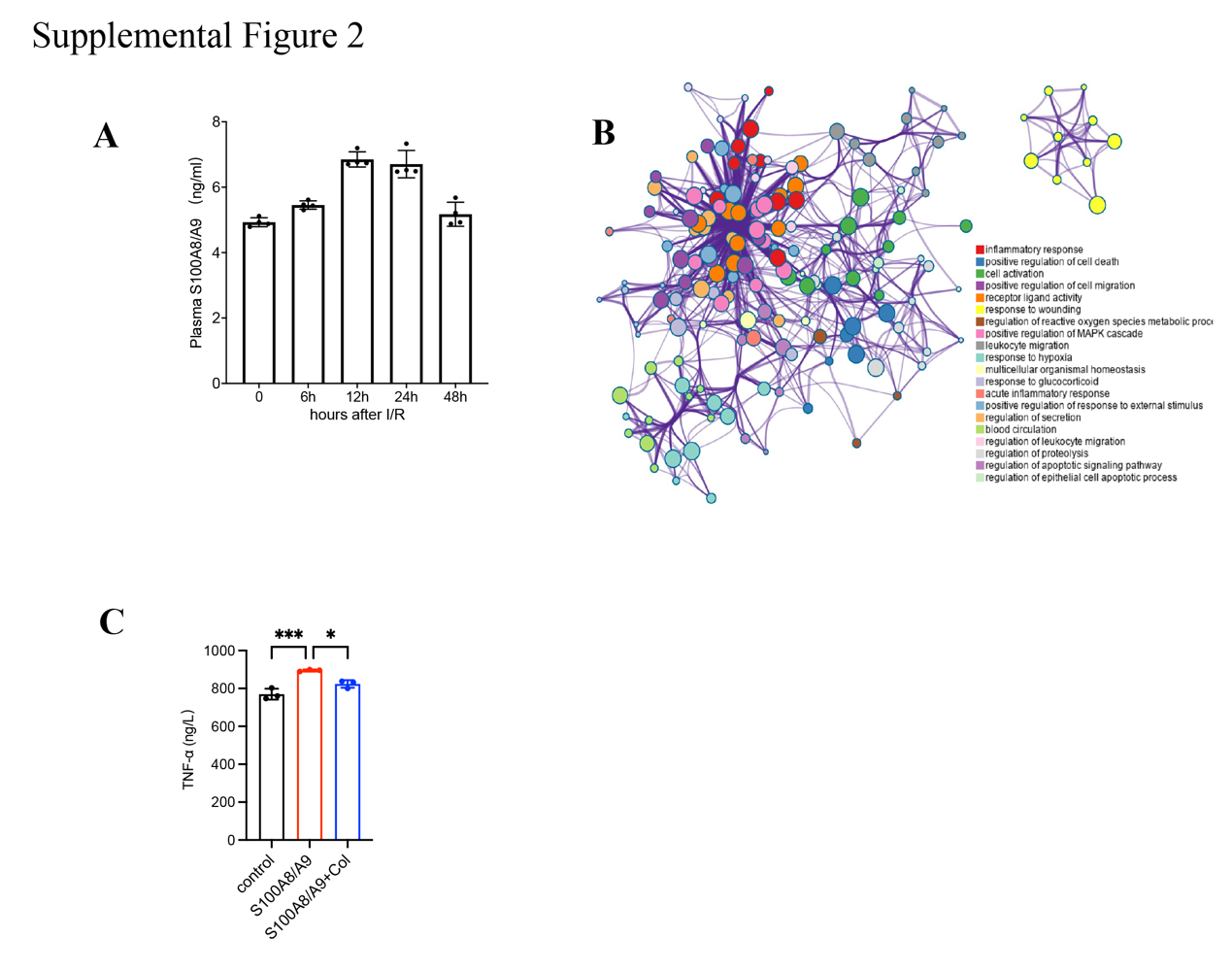
**

**Fig S2.** (**A**) Quantification of S100A8/A9 protein levels (by ELISA) in the plasma of mice at different time points after myocardial I/R (n=4). (**B**) Network of enriched items of 74 genes. (**C**) Quantification of TNF-α protein levels (by ELISA) in the primary neutrophils stimulated with recombinant mouse S100A8/A9 (n=3).
